# The Impact of Political Orientation and Government Change on Public Satisfaction with Food Policy in South Korea

**DOI:** 10.3390/foods13101442

**Published:** 2024-05-07

**Authors:** Sunhyung Min, Sung Ju Cho

**Affiliations:** 1 Center for Agriculture Policy Evaluation, Korea Rural Economic Institute, Naju-si 58217, Republic of Korea; minsh1026@krei.re.kr; 2 Department of Applied Economics, Jeju National University, Jeju-si 63243, Republic of Korea

**Keywords:** food policy, political orientation, government change, policy satisfaction, Consumer Behavior Survey for Foods

## Abstract

This study investigates the relationship between political orientation, government change, and public satisfaction with food policies in the Republic of Korea. Utilizing data from the Consumer Behavior Survey for Foods (CBSF) conducted by the Korea Rural Economic Institute from 2020 to 2021, we employ fixed effects models to examine the impact of political orientation, the presence of a conservative government, and their interactions on satisfaction across various dimensions of food policy. We also analyze the change in satisfaction levels from 2020 to 2021 based on shifts in political orientation. The results reveal complex dynamics between political alignment, government performance, and public perceptions. While conservatives and liberals exhibit higher satisfaction with labeling policies, they show lower satisfaction with safety and redress policies. The presence of a conservative government is associated with higher satisfaction in specific policy areas but lower overall satisfaction. Changes in political orientation significantly influence policy satisfaction, with shifts away from conservatism and towards liberalism leading to decreased satisfaction. The findings highlight the importance of understanding the nuanced preferences of different political groups and the need for responsive and transparent food policy frameworks. This study advances the theoretical understanding of the political economy of policy satisfaction and provides novel policy implications for effective governance.

## 1. Introduction

Food policy plays a pivotal role in shaping public health outcomes, economic well-being, and societal satisfaction. As governments navigate the complex landscape of food safety, pricing, consumer protection, and sustainability, understanding the interplay between political dynamics and public perceptions becomes paramount [1]. This study delves into the nuanced relationship between political orientation, government transitions, and public satisfaction with various dimensions of food policy in the Republic of Korea.

By examining how political orientation and government changes influence satisfaction across key areas of food policy—safety, pricing, redress, education, labeling, and overall perception—this study aims to provide valuable insights for policymakers, researchers, and stakeholders.

Food policy encompasses a wide array of regulations, initiatives, and programs designed to ensure a safe, affordable, and sustainable food system. From setting safety standards and monitoring compliance to implementing pricing mechanisms and providing consumer education, food policy directly impacts the daily lives of citizens [2].

The intersection of political ideology, public trust, and policy satisfaction is a complex domain that requires careful examination. Political orientation can shape individuals’ expectations, priorities, and evaluations of government performance [3]. Van de Walle and Bouckaert (2003) suggest that the relationship between public service performance and trust in government is multifaceted, with factors such as the perception of agencies as part of the government, dominant institutions that shape views of the government, and the criteria citizens use to evaluate the government beyond just performance all playing a role in this dynamic [4]. By analyzing these dynamics through the lens of food policy in the Republic of Korea, this study contributes to a deeper understanding of how political factors shape public perceptions and satisfaction.

The study’s theoretical framework integrates insights from multiple perspectives to provide a comprehensive understanding of the complex relationship between political orientation, government transitions, and public satisfaction with food policies. Drawing upon the expectancy–disconfirmation model [5], we posit that individuals’ satisfaction with government services is influenced by the discrepancy between their expectations and perceived performance. We extend this model by incorporating the role of political orientation as a key factor shaping individuals’ expectations and evaluations of government performance.

Moreover, we draw upon the literature on political trust to theorize how government transitions can influence public perceptions of policy effectiveness and responsiveness [6]. This perspective is further enriched by the party identification theory [7], which suggests that individuals’ loyalty to a particular political party serves as a perceptual screen through which they evaluate government performance and policy outcomes. In the context of food policy, this theory implies that individuals who identify with the ruling party may be more likely to express satisfaction with government actions, while those who identify with the opposition party may be more likely to express dissatisfaction, regardless of the objective effectiveness of the policies in question.

Furthermore, we integrate the policy feedback theory [8] into our framework, recognizing that policies themselves can shape public attitudes and political behavior over time. In the case of food policy, the design and implementation of specific programs and regulations may influence how different groups perceive and respond to government actions. For instance, policies that are seen as benefiting certain regions or socioeconomic groups may generate positive feedback and increase satisfaction among those constituencies, while policies that are perceived as unfair or burdensome may generate negative feedback and decrease satisfaction.

By synthesizing these theoretical perspectives, our study aims to provide a nuanced and holistic understanding of the factors that shape public satisfaction with food policies in the Republic of Korea. This integrated framework allows us to examine the interplay between individual-level factors such as political orientation and party identification, as well as the broader institutional and policy context that influences public perceptions and attitudes over time. Through this approach, we seek to contribute to the literature on political trust, policy feedback, and public opinion, while also generating practical insights for policymakers seeking to design and implement effective and responsive food policies.

By applying this theoretical lens to the analysis of food policy satisfaction in the Republic of Korea, we aim to provide a nuanced understanding of how political orientation and government change interact to shape public perceptions across various dimensions of food policy. This study’s findings not only contribute to the growing body of literature that explores the intricate connections between political factors and policy satisfaction, but also offer valuable insights for policymakers seeking to design effective and responsive food policies in an era of political polarization and public scrutiny.

In recent years, the Republic of Korea has witnessed a surge in public interest and concern regarding food policy issues. High-profile incidents involving food safety violations, price fluctuations, and consumer protection have sparked intense media scrutiny and public debate [9,10,11]. The Korean government has responded with a range of policy initiatives, including stricter regulations on food labeling and advertising, increased investment in food safety inspections, and efforts to stabilize prices of staple foods [12,13,14,15]. However, these efforts have been met with mixed reactions from the public, with some praising the government’s responsiveness and others criticizing the effectiveness and consistency of its actions. This complex and dynamic context underscores the importance of examining the factors that shape public perceptions and satisfaction with food policy in the Republic of Korea.

A key strength of this study is its use of longitudinal data from the Consumer Behavior Survey for Foods (CBSF) conducted by the Korea Rural Economic Institute. This survey provides rich information on public perceptions and satisfaction with various aspects of food policy over a three-year period from 2020 to 2022. Importantly, this period encompasses a significant political transition in the Republic of Korea, allowing us to examine how changes in government affect public attitudes and satisfaction. By leveraging this unique dataset and employing advanced statistical techniques such as fixed effects models and interaction effects, we are able to control for a wide range of individual and household characteristics that may influence policy satisfaction. This approach enhances the robustness and validity of our findings, and sets our study apart from previous research that has relied on cross-sectional data or more limited statistical methods.

The findings of this research hold important implications for policymakers, highlighting the need for responsive, inclusive, and evidence-based approaches to food policy. By understanding the nuanced preferences and expectations of different political groups, governments can better align their policies with public needs and values [16]. Moreover, the study emphasizes the importance of effective communication, transparency, and public engagement in building trust and satisfaction with government actions.

In the following sections, we present the data and methods employed in this study, discuss the key results, and explore their implications for food policy and political dynamics in the Republic of Korea. We then delve into the complexities of the findings, acknowledge the study’s limitations, and offer suggestions for future research. Through this comprehensive analysis, we aim to contribute to the understanding of the intricate relationships between politics, public opinion, and policy satisfaction in the context of food policy.

Building upon the theoretical framework, this study aims to address the following research questions: How do political orientation and government change influence public satisfaction across different dimensions of food policy in the Republic of Korea? How do changes in political orientation over time affect changes in food policy satisfaction? By explicitly addressing these questions, we seek to provide a comprehensive understanding of the complex dynamics between political factors and public perceptions of food policy effectiveness.

## 2. Theoretical Framework

This study aims to investigate the relationship between political orientation, government change, and public satisfaction with food policies in the Republic of Korea. To provide a comprehensive theoretical foundation for our analysis, we integrate insights from various perspectives, including the expectancy–disconfirmation model, political trust theory, party identification theory, and policy feedback theory. This integrated framework allows us to examine the complex interplay between individual-level factors, such as political orientation and party identification, and the broader institutional and policy context that shapes public perceptions and attitudes over time.

We conceptualize an individual’s utility as a function of various factors that contribute to their overall well-being. In the context of food policy, an individual’s utility may be affected by factors such as food safety, price stability, consumer protection, and the overall quality and sustainability of the food system. The relative importance of these factors may vary across individuals depending on their socioeconomic status, cultural background, and personal preferences. Satisfaction can be viewed as a specific component of an individual’s overall utility, representing the degree to which an individual’s expectations or desires are met in a particular domain. In the case of food policy, satisfaction refers to the extent to which citizens perceive the government’s actions and decisions as effective, responsive, and aligned with their interests and values.

Formally, we can express the relationship between satisfaction and utility as:

(1) $$U_{i}=U\left(S_{i},X_{i}\right)$$

where $U_{i}$ denotes individual i’s utility, $S_{i}$ represents individual i’s satisfaction with food policy, and $X_{i}$ represents other factors that affect utility beyond policy satisfaction. This formulation suggests that policy satisfaction is a distinct and integral component of an individual’s overall utility, and changes in satisfaction levels, holding other factors constant, can lead to changes in utility.

Political orientation is a key factor shaping individuals’ expectations, priorities, and evaluations of government performance [3]. In the context of food policy, conservatives, moderates, and liberals may have different preferences and expectations regarding the role of government in regulating food safety, pricing, and consumer protection. These differences in political orientation can influence individuals’ satisfaction with various dimensions of food policy. Changes in government, such as the transition from a liberal to a conservative administration in the Republic of Korea, can signal shifts in policy approaches and priorities [17]. These transitions may lead to variations in public satisfaction, as individuals adjust their expectations and evaluations based on the perceived alignment between their political orientation and the government’s actions.

The impact of political orientation on policy satisfaction may be moderated by government change. For example, conservatives may exhibit higher satisfaction with food policies when a conservative government is in power, while liberals may express greater dissatisfaction during the same period. The interaction between political orientation and government change captures these differential effects and provides insights into the complex dynamics of public opinion formation. In addition to political orientation and government change, various individual and household characteristics may influence policy satisfaction, such as socioeconomic status, consumption patterns, health status, and demographic variables [18].

Drawing upon the expectancy–disconfirmation model [5], we posit that an individual’s satisfaction with food policy (S) is a function of their political orientation (P), the government in power (G), and other relevant factors (Z). The satisfaction function can be expressed as:

(2) $$S_{i}=S\left(P_{i},G,Z_{i}\right)$$

Assuming that individuals aim to maximize their utility, which is partially determined by policy satisfaction, we can derive first-order conditions:

(3) $$\frac{{\partial U}_{i}}{\partial S_{i}}\frac{{\partial S}_{i}}{\partial P_{i}}=0;$$

(4) $$\frac{{\partial U}_{i}}{\partial S_{i}}\frac{{\partial S}_{i}}{\partial G_{i}}=0$$

that relate changes in satisfaction to changes in its determinants.

The theoretical model developed in this section directly informs our empirical specification. The fixed effects model presented in the data and methods section (Equation (1)) is derived from the linearized satisfaction function, with the inclusion of interaction terms and control variables to capture the nuances of the relationships between political orientation, government change, and policy satisfaction. The choice of variables in our empirical analysis is grounded in the theoretical framework, with the inclusion of political orientation, government change, and their interaction term justified by the expectancy–disconfirmation model and the theories of political trust and party identification.

Based on the theoretical framework, we hypothesize that political orientation will have a significant impact on food policy satisfaction, with conservatives, moderates, and liberals exhibiting different levels of satisfaction across various policy dimensions. We also expect that government change will influence satisfaction levels, with the direction and magnitude of the effect varying depending on the political orientation of the individual and the specific policy domain.

While we conceptualize satisfaction as a component of utility, it is important to acknowledge the potential conceptual differences between the two constructs. Utility is a broader concept that encompasses various aspects of an individual’s well-being, while satisfaction focuses specifically on the evaluation of government performance in a particular policy domain. We recognize this limitation and interpret our findings with appropriate caution. Our theoretical model also makes several simplifying assumptions, such as the linearity of the satisfaction function and the focus on a limited set of determinants. These assumptions are necessary for tractability and empirical estimation, but they may not fully capture the complexity of the real-world relationships between political orientation, government change, and policy satisfaction. We acknowledge these limitations and discuss their implications for the interpretation of our results.

The relationship between political orientation and policy satisfaction may be subject to endogeneity concerns, as individuals’ satisfaction levels may influence their political attitudes and affiliations over time. While our panel data approach and the use of fixed effects help mitigate some of these concerns, we cannot entirely rule out the possibility of reverse causality or omitted variable bias. We address this limitation by interpreting our results as associations rather than causal effects and by discussing the potential endogeneity issues in the context of our findings.

By incorporating the concept of utility and the expectancy–disconfirmation model into our analysis of policy satisfaction, we bridge the gap between economic theory and political science perspectives. This interdisciplinary approach allows for a more comprehensive understanding of the factors that shape public opinion and policy evaluation in the context of food policy.

## 3. Materials and Methods

### 3.1. Materials

This study utilizes data from the Consumer Behavior Survey for Foods (CBSF) conducted by the Korea Rural Economic Institute. The survey provides valuable insights into public perceptions and satisfaction with various aspects of food policy in the Republic of Korea. The data covers the period from 2020 to 2022, capturing a significant political transition from a liberal to a conservative government.

Table 1 presents the distribution of political orientation across the three years covered in the study. As shown in the table, there is a notable shift in the distribution of political orientation over time, with an increase in the proportion of conservatives and a decrease in the proportion of liberals from 2020 to 2022. This shift aligns with the political transition captured in the data, providing context for the subsequent analysis.

The CBSF measures satisfaction across six key policy dimensions: (1) Food Safety Policy, (2) Food Fair Pricing/Trade Policy, (3) Food Damage Compensation Policy, (4) Food-related Education and Promotion Policy, (5) Food Labeling Policy, and (6) Overall Satisfaction. These dimensions encompass a wide range of food policy aspects, allowing for a comprehensive assessment of public perceptions.

### 3.2. Methods

To analyze the relationship between political orientation, government change, and policy satisfaction, we employed a fixed effects model. The use of fixed effects models is particularly suitable for analyzing panel data, as it allows for controlling unobserved time-invariant factors that may influence the dependent variable [19]. In the context of food policy satisfaction, employing fixed effects models enables us to account for individual-specific characteristics that remain constant over time, such as cultural background or long-standing political beliefs, which may affect perceptions of policy effectiveness. By isolating these time-invariant factors, we can obtain more precise estimates of the relationships between political orientation, government change, and policy satisfaction. This approach has been used in previous studies investigating consumer preferences for food quality. For example, Felderhoff et al. (2020) employ fixed effects models to examine the factors driving consumer satisfaction with beef quality, accounting for consumer-specific fixed effects to control for unobserved heterogeneity in preferences [20].

Our model can be expressed as follows:

(5) $$SATIS_{it}=\delta_{1}Pol_{it}+\delta_{2}Govt_{t}+\delta_{3}\left(Pol_{it}\times Govt_{t}\right)+\beta X_{it}+\mu_{i}+\varepsilon_{it},$$

where $SATIS_{it}$ is the satisfaction score of individual i at time t for a specific policy dimension, $Pol_{it}$ represents the political orientation of individual i at time t, and $Govt_{t}$ is a dummy variable indicating the government in power at time t, with 1 for a conservative government and 0 otherwise. The interaction term $Pol_{it}\times Govt_{t}$ captures the differential impact of political orientation under different government regimes. $\mu_{i}$ and $\varepsilon_{it}$ represent the individual fixed effects and the error term, respectively.

The model also includes a vector of control variables $X_{it}$, such as personal income, food away from home (FAFH) expenses, health status (overweight and underweight), age, sex, education, marital status, household income, household food expenses, number of young and adult household members, and residential area (rural or urban). These variables account for individual and household characteristics that may influence policy satisfaction [18].

Table 2 displays the changes in political orientation from 2020 to 2021. The table reveals substantial shifts in political orientation during this period, with notable movements from liberal to moderate and conservative orientations, as well as from moderate to conservative and liberal orientations.

To investigate the relationship between changes in political orientation and changes in policy satisfaction from 2020 to 2021, we estimated a model using the change in satisfaction scores ($\Delta SATIS_{i}$) as the dependent variable. This allowed us to examine how shifts in political orientation are associated with changes in policy satisfaction over time.

The model is specified as:

(6) $$SATIS_{i}=\sum_{j=1}^{J}\gamma_{j}Change_{i,j}+{\beta X}_{i}+\varepsilon_{i},$$

where $Change_{i,j}$ represents the change in political orientation of individual i from one category to another, with J being the total number of possible changes. The coefficients $\gamma_{j}$ capture the effect of each type of political orientation change on policy satisfaction, relative to a baseline category. The analysis employs robust standard errors to account for potential heteroskedasticity in the data. The models include province fixed effects to control for regional variations in policy satisfaction.

## 4. Results

The estimation results from the first model, shown in Table 3, reveal the nuanced impact of political orientation, government presence, and their interactions on satisfaction across different food policy dimensions.

Regarding political orientation, conservatives exhibit significantly higher satisfaction with labeling policies compared to those without a stated orientation. This suggests that conservatives may place a higher value on transparency and consumer information. However, the negative coefficient for the safety policy area indicates that conservatives may perceive current safety policies as insufficient or misaligned with their expectations.

Moderates show higher satisfaction with pricing policies but significantly lower satisfaction with redress mechanisms. This implies that moderates appreciate efforts to ensure fair pricing but may view compensation policies as inadequate or ineffective.

Liberals, on the other hand, display significantly lower satisfaction with both safety and redress policies. This critical stance suggests that liberals perceive shortcomings in the effectiveness of these policy areas. However, like conservatives, liberals exhibit higher satisfaction with labeling policies, indicating a shared value for transparency in food information across political orientations.

The presence of a conservative government (Govt = 1) is associated with higher satisfaction in pricing and labeling policies but significantly lower overall satisfaction. This finding suggests that while specific policy implementations under the conservative government may be well-received, there is broader discontent with the government’s overall approach to food policy.

The interaction terms between political orientation and government presence yield notable results. Conservatives show significant negative interactions, particularly in pricing and labeling policies, under a conservative government. This indicates that conservative satisfaction decreases in these areas when a conservative government is in power, contrary to expectations. It suggests a potential disillusionment or heightened expectations among conservatives when their preferred government is in office.

Moderates exhibit a positive interaction effect for redress policies under a conservative government, suggesting that changes made by the conservative government align somewhat with moderate views on compensation policies.

Liberals, surprisingly, also show positive interaction effects for redress and overall satisfaction under a conservative government. This implies that certain policy measures introduced by the conservative government may unexpectedly align with liberal preferences or exceed their expectations in these domains.

The results highlight the complex dynamics between political orientation, government actions, and policy satisfaction. The findings suggest that political alignment does not guarantee higher satisfaction, as individuals may have specific expectations that go beyond broad ideological labels [21,22]. The variation in satisfaction across policy areas underscores the importance of considering policy specificity, as individuals’ preferences and evaluations can differ significantly based on the policy domain [23,24].

Moreover, the overall lower satisfaction under a conservative government, despite higher satisfaction in specific areas, points to a broader discontent with government performance. This emphasizes the need for comprehensive policy evaluation and responsiveness to public needs [6,25].

The control variables in the first model provide valuable insights into the factors influencing food policy satisfaction. Personal income shows significant negative coefficients for pricing, labeling, and overall satisfaction, suggesting that individuals with higher incomes tend to be more critical of these policy areas. This may be due to higher expectations or greater awareness of policy shortcomings among affluent individuals [26]. On the other hand, food away from home (FAFH) expenses exhibit significant positive coefficients across all policy dimensions, indicating that individuals who frequently dine out have higher satisfaction with food policies. This finding highlights the importance of considering lifestyle factors and consumption patterns when assessing public perceptions of food policies [27,28,29,30].

Health status also plays a role in shaping policy satisfaction. Being overweight is associated with significantly higher overall satisfaction, suggesting that overweight individuals may have different priorities or expectations regarding food policies compared to those with normal weight [31]. In contrast, being underweight is associated with lower satisfaction with redress policies, indicating that underweight individuals may perceive inadequacies in compensation mechanisms for food-related issues.

Household income and household food expenses show mixed results, with household income having a negative association with safety and overall satisfaction, while household food expenses are positively related to education policies. These findings suggest that household economic factors influence perceptions of specific policy areas differently, emphasizing the need for targeted policy approaches that consider the diverse needs and concerns of households [32].

The second model, with results shown in Table 4, examines the relationship between changes in political orientation and changes in policy satisfaction from 2020 to 2021.

The results highlight a significant decrease in satisfaction across almost all policy areas for individuals who changed their political orientation, particularly those moving away from a conservative stance (C → M, C → L) and toward a liberal orientation (Z → L), compared to those without a stated orientation in both periods. This finding suggests a strong reaction against the conservative government’s food policies among individuals shifting away from conservatism.

Notably, individuals transitioning from a conservative to a liberal orientation (C → L) exhibit the largest decreases in satisfaction across all policy dimensions, with significant negative coefficients. This implies a notable disappointment with the conservative government’s food policies among those who underwent a drastic shift in political orientation.

Similarly, individuals moving from a moderate to a liberal orientation (M → L) also show significant decreases in satisfaction across most policy areas. This finding suggests that the conservative government’s policies failed to meet the expectations of individuals who previously held moderate views but shifted towards a more liberal stance.

Interestingly, individuals who maintained a liberal orientation (L → L) also exhibited decreases in satisfaction, particularly in the pricing policy area. This implies that even among consistently liberal individuals, there was growing dissatisfaction with the conservative government’s approach to food pricing policies.

The control variables in the second model reinforce the findings from the first model. Higher personal income is associated with decreased satisfaction, while increased FAFH expenses relate to higher satisfaction in specific areas. The more pronounced negative impact of being overweight suggests growing dissatisfaction with the effectiveness of food policies in addressing health and nutrition concerns from 2020 to 2021.

The presence of young household members is associated with increased satisfaction in most policy areas, suggesting that households with children or younger members may have perceived improvements in food policies from 2020 to 2021. This finding highlights the importance of considering the unique needs and preferences of different household compositions when designing and evaluating food policies.

These findings underscore the significant impact of political orientation changes on food policy satisfaction. The results suggest that political shifts reflect deep-seated concerns and expectations among the populace regarding food policy effectiveness, transparency, and responsiveness to health and economic needs. Economic factors, lifestyle choices, and health considerations also play crucial roles in shaping individuals’ perceptions of food policies [27,28,29,30].

## 5. Discussion

The analysis presented in this study reveals the complex interplay between political orientation, government transitions, and public satisfaction with food policies in the Republic of Korea. The findings highlight that political alignment does not always translate into higher satisfaction, as individuals’ preferences and expectations are nuanced and multifaceted.

The results underscore the importance of understanding the specific concerns and priorities of different political groups. Policymakers must navigate the complexities of public opinion and strive to develop policies that address the diverse needs of the population. The varying satisfaction levels across policy dimensions emphasize the need for a comprehensive approach that considers the unique challenges and opportunities in each area.

The study also sheds light on the role of expectations in shaping policy satisfaction. The findings suggest that both policy effectiveness and the management of public expectations are crucial for improving satisfaction with government policies [33,34]. Governments must not only deliver tangible results but also effectively communicate their efforts and engage with the public to build trust and understanding.

The impact of political orientation changes on policy satisfaction highlights the dynamic nature of public opinion. As individuals’ political stances evolve, their perceptions and evaluations of government policies also shift. This underscores the importance of continuous monitoring and responsiveness to changes in public sentiment [35,36]. Governments must be attuned to the evolving needs and concerns of the population and adapt their policies accordingly.

The findings underscore the importance of considering the nuanced preferences of different political and demographic groups when designing and evaluating food policies. Conservatives exhibited higher satisfaction with labeling policies, suggesting a preference for transparency, while liberals displayed lower satisfaction with safety and redress policies, indicating concerns about the adequacy of current measures. Additionally, the presence of young household members and being married were associated with higher satisfaction in certain policy areas, highlighting the importance of considering the unique needs of different household types.

Moreover, the study emphasizes the significance of economic factors, lifestyle choices, and health considerations in shaping individuals’ perceptions of food policies. Policymakers must consider the broader socioeconomic context and how it influences public satisfaction [32]. Addressing issues such as income disparities, changing consumption patterns, and public health challenges is crucial for developing effective and equitable food policies [37].

The findings of this study contribute to the growing body of literature on the intersection of politics, public opinion, and policy satisfaction. The analysis provides valuable insights into the context of the Republic of Korea, highlighting the unique dynamics at play in the realm of food policy. Future research could build upon these findings by exploring additional policy domains, examining longer time periods, or conducting comparative studies across different countries or regions.

## 6. Conclusions

This study provides a comprehensive analysis of the relationship between political orientation, government transitions, and public satisfaction with food policies in the Republic of Korea. By leveraging longitudinal data from the Consumer Behavior Survey for Foods (CBSF) and employing advanced statistical techniques, we uncover the complex interplay of political dynamics, individual preferences, and political orientation in shaping public opinion.

Our findings make several important contributions to the literature on the political determinants of policy satisfaction. First, we provide empirical evidence on how political orientation and government changes influence satisfaction across various dimensions of food policy, extending previous research that has primarily focused on broader measures of political trust and satisfaction. Second, by examining satisfaction at the level of specific policy domains, we highlight the importance of considering policy specificity when analyzing the relationship between political factors and public attitudes. Third, our study underscores the dynamic nature of public opinion, showing how changes in political orientation over time can significantly impact policy satisfaction.

The results of this study also have significant policy implications. Our findings underscore the need for policymakers to be attuned to the diverse preferences and expectations of different political groups when designing and implementing food policies. By understanding how political orientation shapes individuals’ evaluations of policy effectiveness and responsiveness, policymakers can develop more targeted communication strategies to build trust and support among various constituencies. Moreover, our results highlight the importance of policy consistency and continuity, even in the face of political transitions, to maintain public confidence and satisfaction.

The results of this study indicate the need for differentiated food policy approaches based on government type. For liberal governments, it may be necessary to focus on strengthening food safety regulations and expanding consumer redress systems. Conservative governments, on the other hand, could prioritize improving food labeling systems and promoting consumer education for healthy food choices. Meanwhile, policies that can garner support from the majority of the population, regardless of political orientation, include enhancing food safety management systems and stabilizing food prices.

To translate these implications into concrete policy actions, we propose several recommendations. First, governments should establish institutional mechanisms for regular public consultation and participation in the policymaking process, such as citizen advisory committees or online feedback platforms. This can help ensure that diverse political perspectives are considered and incorporated into policy decisions. Second, policymakers should develop differentiated policy communication and outreach strategies tailored to the specific concerns and priorities of different political groups. For example, emphasizing transparency and consumer information may be particularly effective for engaging conservative constituents, while highlighting safety and redress mechanisms may resonate more with liberal audiences. Third, governments should strive to maintain a stable and predictable policy environment by setting clear long-term goals and avoiding sudden shifts in policy priorities or implementation approaches. This can help build public trust and mitigate the negative impact of political transitions on policy satisfaction.

However, it is important to acknowledge the limitations of this study and suggest avenues for future research. While our analysis covers a significant political transition in the Republic of Korea, the relatively short time period (2020–2022) and the focus on a single country context may limit the generalizability of our findings. Future studies could extend our analysis by examining longer time horizons and conducting cross-national comparisons to identify common patterns and context-specific factors shaping the relationship between political variables and policy satisfaction. Additionally, our reliance on survey data may be subject to self-reporting biases and limitations in capturing the full complexity of individuals’ opinions and experiences. Complementing survey-based analyses with qualitative methods, such as in-depth interviews or focus group discussions, could provide a richer understanding of the mechanisms linking political orientation, government performance, and public attitudes.

Moreover, while our study focuses on the specific domain of food policy, our findings have broader implications for understanding the relationship between politics and policy satisfaction across different contexts. The political dynamics and public opinion patterns we uncover in the Republic of Korea case may offer valuable insights for other countries grappling with similar challenges of ensuring public trust and satisfaction in an era of political polarization and transition. By situating our results within the wider international literature on political trust, policy feedback, and public opinion, we aim to contribute to ongoing debates and inform future research on the political foundations of effective and responsive policymaking.

In conclusion, this study advances our understanding of the complex interplay between political orientation, government transitions, and public satisfaction with food policies in the Republic of Korea. Our findings highlight the importance of considering the nuanced preferences of different political groups and the need for responsive and transparent policy frameworks in shaping public opinion. By proposing concrete policy recommendations and identifying avenues for future research, we seek to provide valuable insights for policymakers and scholars alike. Ultimately, promoting public satisfaction and trust in food policies requires a sustained commitment to evidence-based, inclusive, and adaptable policymaking that is attuned to the evolving needs and concerns of the population.

## Figures and Tables

**Table 1:** Distribution of political orientation by year.

Year	No Ans. /Don’t Know	Conservative	Moderate	Liberal	Total
2020	758	721	854	1002	3335
2021	573	722	821	693	2809
2022	673	793	741	740	2947
Total	2004	2236	2416	2435	9091

**Table 2:** Changes in political orientation from 2020 to 2021.

Political Orientation in 2020	Political Orientation in 2021				
	No Ans./ Don’t Know	Conservative	Moderate	Liberal	Total
No answer/don’t know	1226	300	518	374	2418
Conservative	339	1013	320	191	1863
Moderate	560	437	762	467	2226
Liberal	551	308	774	951	2584
Total	2676	2058	2374	1983	9091

**Table 3:** Impact of political orientation and government change on food policy satisfaction.

	(1)	(2)	(3)	(4)	(5)	(6)
	Safety	Pricing	Redress	Education	Labeling	Overall
Pol:						
Conservative	−0.532	1.420	0.234	−0.071	2.130 **	−0.477
	(0.839)	(0.920)	(0.857)	(0.839)	(0.885)	(0.655)
Moderate	−0.331	1.529 *	−1.345 *	−0.294	2.293 ***	−0.429
	(0.794)	(0.847)	(0.810)	(0.785)	(0.838)	(0.616)
Liberal	−1.606 **	0.163	−1.748 **	−0.836	1.930 **	−0.689
	(0.747)	(0.823)	(0.773)	(0.740)	(0.793)	(0.572)
Govt = 1	−0.428	1.317 *	−0.963	−0.538	2.748 ***	−1.498 ***
	(0.702)	(0.760)	(0.735)	(0.685)	(0.736)	(0.557)
Pol × Govt:						
Conservative × Govt = 1	−0.893	−2.789 ***	−1.210	−0.589	−2.829 ***	−0.601
	(0.868)	(0.953)	(0.936)	(0.867)	(0.926)	(0.692)
Moderate × Govt = 1	0.190	−2.256 **	1.587 *	0.188	−0.984	0.320
	(0.896)	(0.963)	(0.949)	(0.898)	(0.969)	(0.733)
Liberal × Govt = 1	1.686 *	−0.891	2.169 **	0.989	−1.657 *	1.159 *
	(0.862)	(0.975)	(0.934)	(0.890)	(0.975)	(0.697)
Year = 2021	0.248	−0.021	−0.468	−0.558 **	−0.818 ***	−0.029
	(0.264)	(0.290)	(0.289)	(0.274)	(0.315)	(0.243)
Personal income	0.000	−0.004 *	−0.003	−0.002	−0.004 *	−0.004 ***
	(0.002)	(0.002)	(0.002)	(0.002)	(0.002)	(0.001)
FAFH expense	0.127 ***	0.118 ***	0.108 ***	0.139 ***	0.097 ***	0.079 ***
	(0.023)	(0.027)	(0.025)	(0.025)	(0.026)	(0.019)
Overweight	0.340	−0.154	−0.038	0.159	−0.135	1.237 ***
	(0.569)	(0.629)	(0.597)	(0.578)	(0.639)	(0.471)
Underweight	−0.345	−1.489	−2.440 *	−1.755	−0.704	−1.363
	(1.095)	(1.249)	(1.351)	(1.233)	(1.217)	(1.139)
HH income	−0.003 **	−0.002	−0.002	−0.002	−0.001	−0.001
	(0.001)	(0.002)	(0.002)	(0.001)	(0.002)	(0.001)
HH food expense	−0.002	−0.006	0.006	0.014 **	−0.013 *	−0.005
	(0.006)	(0.007)	(0.007)	(0.007)	(0.007)	(0.006)
Constant	77.485 ***	75.089 ***	76.692 ***	77.091 ***	73.541 ***	78.910 ***
	(0.921)	(0.987)	(0.967)	(0.931)	(0.989)	(0.754)
Observations	8087	8087	8087	8087	8087	8087
Number of clusters	2828	2828	2828	2828	2828	2828
Adj. R-squared	0.427	0.408	0.389	0.427	0.396	0.452

Notes: The baseline category for political orientation is “no answer/don’t know”. Clustered robust standard errors are in parentheses (* *p* < 0.10, ** *p* < 0.05, *** *p* < 0.01).

**Table 4:** Changes in policy satisfaction from 2020 to 2021 by political orientation shifts.

	(1)	(2)	(3)	(4)	(5)	(6)
	ΔSafety	ΔPricing	ΔRedress	ΔEducation	ΔLabeling	ΔOverall
Pol changes:						
C → C	−0.330	−4.307 ***	−3.119 **	−1.498	−4.664 ***	−1.510
	(1.443)	(1.598)	(1.553)	(1.509)	(1.627)	(1.117)
C → M	−0.665	−7.398 ***	−5.429 ***	−4.152 **	−4.827 **	−3.501 ***
	(1.738)	(1.925)	(1.871)	(1.818)	(1.960)	(1.346)
C → L	−4.512 **	−9.038 ***	−5.480 **	−6.956 ***	−7.511 ***	−0.075
	(2.084)	(2.308)	(2.243)	(2.179)	(2.350)	(1.613)
C → Z	−0.816	−5.849 ***	−3.600 *	−0.489	−4.455 **	−1.815
	(1.998)	(2.212)	(2.150)	(2.089)	(2.253)	(1.547)
M → C	−1.655	−6.864 ***	−3.254 *	−2.620	−5.741 ***	−2.006
	(1.605)	(1.778)	(1.728)	(1.679)	(1.810)	(1.243)
M → M	−0.994	−5.878 ***	−2.099	−2.754 *	−4.561 ***	−1.096
	(1.377)	(1.525)	(1.482)	(1.440)	(1.553)	(1.066)
M → L	1.758	−2.670	−1.030	−0.238	−2.827	−0.949
	(1.550)	(1.717)	(1.668)	(1.621)	(1.748)	(1.200)
M → Z	1.676	−2.883	−0.368	0.969	−6.476 ***	−0.815
	(1.605)	(1.778)	(1.728)	(1.679)	(1.810)	(1.243)
L → C	0.597	−5.746 ***	−1.349	−2.471	−3.209	−1.438
	(1.773)	(1.963)	(1.908)	(1.854)	(1.999)	(1.373)
L → M	2.300 *	−2.491	−0.270	0.153	−2.147	−1.095
	(1.382)	(1.530)	(1.487)	(1.445)	(1.558)	(1.070)
L → L	−0.153	−6.023 ***	−2.712 *	−2.242	−5.655 ***	−1.488
	(1.338)	(1.482)	(1.440)	(1.399)	(1.509)	(1.036)
L → Z	1.424	−3.139 *	−0.446	−0.300	−3.368 *	−0.918
	(1.635)	(1.811)	(1.760)	(1.710)	(1.844)	(1.266)
Z → C	1.021	−1.340	−1.454	0.238	−1.776	0.849
	(1.915)	(2.121)	(2.062)	(2.003)	(2.160)	(1.483)
Z → M	0.048	−3.492 **	−2.031	−0.590	0.366	−1.418
	(1.545)	(1.711)	(1.663)	(1.616)	(1.742)	(1.196)
Z → L	3.403 **	−2.872	0.783	0.517	0.875	1.173
	(1.671)	(1.850)	(1.798)	(1.747)	(1.884)	(1.294)
Controls:						
Personal income	−0.007 **	−0.005	−0.011 ***	−0.010 ***	−0.007 **	−0.006 ***
	(0.003)	(0.003)	(0.003)	(0.003)	(0.003)	(0.002)
FAFH expense	0.102 ***	0.049	0.102 ***	0.066 *	0.050	−0.005
	(0.035)	(0.039)	(0.038)	(0.037)	(0.040)	(0.027)
Overweight	−1.533 **	−1.834 **	−2.868 ***	−2.512 ***	−2.368 ***	−2.049 ***
	(0.760)	(0.841)	(0.817)	(0.794)	(0.857)	(0.588)
Underweight	−0.220	0.253	−0.083	0.296	−1.129	−3.209 *
	(2.322)	(2.571)	(2.499)	(2.429)	(2.619)	(1.798)
Age	0.047	0.044	0.043	0.056	0.009	−0.006
	(0.036)	(0.039)	(0.038)	(0.037)	(0.040)	(0.028)
Sex (1 = female, 0 = male)	0.029	−0.116	−0.015	−1.231	−0.092	−0.733
	(0.969)	(1.073)	(1.043)	(1.014)	(1.093)	(0.750)
Education	−0.639	−0.754	−0.508	−0.803	−0.600	0.030
	(0.547)	(0.605)	(0.588)	(0.572)	(0.616)	(0.423)
Married	0.378	−0.435	0.663	1.121	−1.179	−0.283
	(0.841)	(0.931)	(0.905)	(0.879)	(0.948)	(0.651)
HH income	−0.002	0.006 **	0.001	0.003	0.001	0.001
	(0.002)	(0.002)	(0.002)	(0.002)	(0.003)	(0.002)
HH food expense	0.004	0.001	0.004	0.019 *	−0.026 **	−0.004
	(0.010)	(0.011)	(0.011)	(0.010)	(0.011)	(0.008)
Number of young	1.492 **	1.738 **	1.462 **	1.754 ***	1.238 *	−0.098
	(0.628)	(0.695)	(0.676)	(0.657)	(0.708)	(0.486)
Number of adults	0.398	−0.263	−0.204	−0.436	0.715	−0.473
	(0.488)	(0.540)	(0.525)	(0.510)	(0.550)	(0.378)
Rural	1.558 *	0.855	0.346	0.349	0.915	0.544
	(0.804)	(0.891)	(0.866)	(0.841)	(0.907)	(0.623)
Constant	−5.097	0.080	−0.597	−0.060	6.839	2.067
	(3.794)	(4.201)	(4.083)	(3.968)	(4.278)	(2.937)
Observations	2725	2725	2725	2725	2725	2725
Adj. R-squared	0.077	0.047	0.042	0.043	0.072	0.027

Notes: C, M, L, and Z indicate conservative, moderate, liberal, and no answer/don’t know, respectively. The baseline category for political orientation changes is Z → Z (“no answer/don’t know” in both periods). Clustered robust standard errors are in parentheses (* *p* < 0.10, ** *p* < 0.05, *** *p* < 0.01). Province fixed effects are included.

## Data Availability

The data used in this article are openly available and accessible at https://www.krei.re.kr/foodSurvey/index.do, accessed on 1 November 2023.
